# Efficient recellularisation of decellularised whole-liver grafts using biliary tree and foetal hepatocytes

**DOI:** 10.1038/srep35887

**Published:** 2016-10-21

**Authors:** Satoshi Ogiso, Kentaro Yasuchika, Ken Fukumitsu, Takamichi Ishii, Hidenobu Kojima, Yuya Miyauchi, Ryoya Yamaoka, Junji Komori, Hokahiro Katayama, Takayuki Kawai, Elena Yukie Yoshitoshi, Sadahiko Kita, Katsutaro Yasuda, Shinji Uemoto

**Affiliations:** 1 Department of Surgery, Graduate School of Medicine, Kyoto University, Kyoto, Japan

## Abstract

A whole-organ regeneration approach, using a decellularised xenogeneic liver as a scaffold for the construction of a transplantable liver was recently reported. Deriving suitable scaffolds was the first step towards clinical application; however, effective recellularisation remains to be achieved. This report presents a strategy for the improvement of the recellularisation process, using novel cell-seeding technique and cell source. We evaluated recellularised liver grafts repopulated through the portal vein or the biliary duct with mice adult hepatocytes or E14.5 foetal hepatocytes. More than 80% of the cells seeded through the biliary tree entered the parenchyma beyond the ductule-lining matrix barrier and distributed throughout the liver lobule. In contrast, about 20% of the cells seeded through the portal tree entered the parenchyma. The gene expression levels of foetal hepatocyte albumin, glucose 6-phosphatase, transferrin, cytokeratin 19, and gamma-glutamyl transpeptidase were increased in three-dimensional cultures in the native liver-derived scaffolds, and the activation of liver detoxification enzymes and formation of biliary duct-like structures were supported. The metabolic functions of liver grafts recellularised with different cell types were similar. These results suggest that biliary tree cell-seeding approach is promising, and that liver progenitor cells represent a good cell source candidate.

Liver transplantation is the ultimate treatment for end-stage liver failure; however, donor organ shortage presents a serious problem. The development of alternative options for liver replacement is necessary, and cell-based therapy and tissue regeneration have been studied, but satisfactory outcomes have not yet been achieved. Therefore, the construction of a fully functional transplantable liver is needed. Three factors are considered essential for organ engineering: tissue-specific cells, scaffolding biomaterials, and the appropriate environment for the promotion of tissue formation^1^. The construction of a three-dimensional (3D) organ scaffold with a complex structure is technically challenging, and the lack of adequate scaffolds has impeded whole-organ engineering. Recently, a decellularisation technique was successfully adapted for the generation of 3D solid organ scaffolds, such as heart^2^, lung^3^, liver^4^,^5^, and kidney^6^. During decellularisation, cells are removed from the extracellular matrix (ECM) of the native organ, and a 3D organ scaffold is produced, while retaining tissue-specific 3D ultrastructure and composition, intact vascular networks for nutrient and gas exchange, and functional and structural molecules^4^. These features have made decellularisation a near-ideal choice for the fabrication of suitable scaffolds for whole-organ regeneration. Several studies have reported whole-liver regeneration using decellularised liver scaffolds, and this process consists of multiple steps—the decellularisation of a donor liver, repopulation of the decellularised liver scaffold (recellularisation), re-establishment of a functional liver graft through an *in vitro* culture, and the implantation of the engineered liver *in vivo*^4^,^5^,^7^,^8^. Obtaining suitable scaffolds represents the first step in the process; however, in order for this process to be applicable in a clinical setting, effective recellularisation needs to be achieved prior to the implantation, together with the reestablishment of the parenchyma, vasculature, and supporting components. Establishing efficient seeding methods and finding clinically relevant, renewable cell sources are the main issues that require solutions to improve previous recellularisation strategies that mainly utilise the portal vein (PV) as a seeding route^4^,^5^,^7^ and primary hepatocytes as a cell source^4^,^7^. Here, we have investigated a new seeding approach, using the biliary tree as a parenchyma repopulation pathway, and foetal hepatocytes as a cell source.

## Results

### Characterisation of decellularised liver scaffold

**Figure 1:**
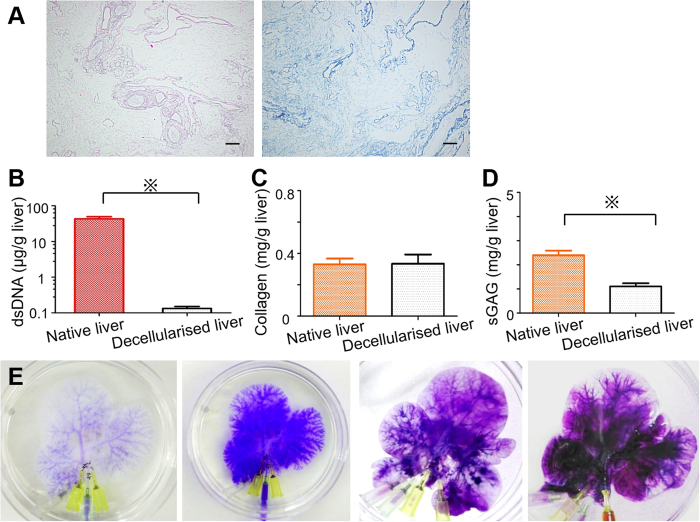
Characterisation of decellularised liver scaffolds. (**A**) H&E and Masson’s trichrome staining showed no remaining nuclei or cytoplasm in the scaffolds. (**B**) Quantification of double-stranded DNA revealed that the decellularisation procedure eliminated more than 99% DNA content in the native livers. (**C,D**) Quantification of ECM components indicated that a 100% of the fibrillary collagen (**C**) and 46% of the sulfated glycosaminoglycans (sGAG) (**D**) were retained after decellularisation. All error bars represent standard error of mean (SEM; n = 3) (^※^p < 0.05). (**E**) Intrahepatic vasculature, demonstrating the integrity of the portal tree (early phase; (E, leftmost panel), late phase; (E, second panel from the left)), arterial tree (E, second panel from the right), and biliary tree (E, rightmost panel).

Translucent acellular scaffold, which retained the gross anatomical features of the liver, was generated after 24 h of decellularisation procedure. Histological evaluation of the decellularised liver scaffolds showed the absence of nuclei or cytoplasmic components in the scaffolds (Fig. 1A). Residual DNA content in the decellularised liver scaffolds was shown to be 0.132 ± 0.017 μg/g liver, demonstrating that more than 99% of the total DNA content was removed from the native liver tissue (43.4 ± 6.42 μg/g liver, p < 0.01) (Fig. 1B).

Quantification of ECM components indicated that a 100% of the fibrillary collagen of the native liver was retained after decellularisation (0.33 ± 0.04 mg/g of native liver, compared with 0.33 ± 0.06 mg/g of decellularised liver) (Fig. 1C). Additionally, 46% of the sulfated glycosaminoglycans were retained after decellularisation (2.4 ± 0.2 mg/g, in the native liver, compared with 1.1 ± 0.1 mg/g of the decellularised liver) (Fig. 1D). Liver vasculature dyeing showed that all three vascular systems, portal, arterial, and biliary trees, were intact after the decellularisation (Fig. 1E), allowing recellularisation and perfusion culture. Furthermore, the blue dye was observed to flow from the portal main vessels to the smaller capillaries (Fig. 1E, leftmost panel), colouring the liver parenchyma next (Fig. 1E, second panel from the left), and it was finally drained into the central venous system, indicating that the decellularised liver scaffold retained the polarity of perfusion from the Glissonian area to the central area.

### Seeding route for the efficient repopulation of scaffold: portal vein versus biliary duct

**Figure 2:**
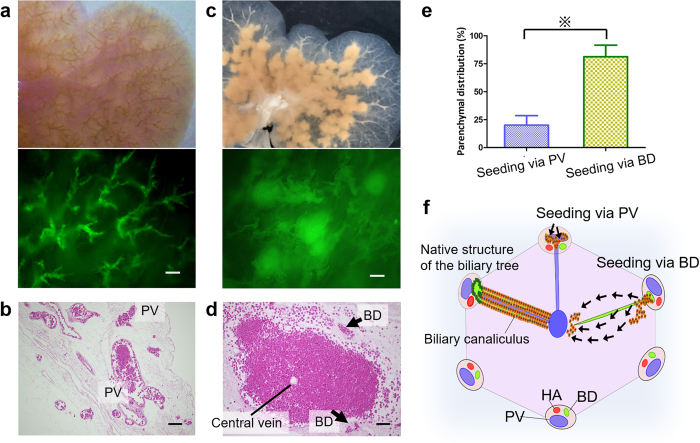
Cell distribution analyses. (**a**) Gross and fluorescence microscopic observations 3 h after portal vein (PV)-seeding. (**b**) H&E staining of recellularised liver sections after PV-seeding, revealing that the majority of cells remained within portal branches at 60 h. (**c**) Gross and fluorescence microscopic observations 3 h after biliary duct (BD)-seeding. (**d**) H&E staining of recellularised liver sections after BD-seeding, revealing massive cell distribution around central veins. (**e**) The percentage of cells distributed in the parenchyma after BD-seeding or PV-seeding. (**f**) Schematic illustration of the migration of seeded cells in the liver lobule (HA, hepatic artery). Scale bars: 250 mm (**a,c**) and 100 μm (**b,d**). All error bars represent standard error of mean (SEM; n = 3) (※ p < 0.05).

The engraftment efficiency of mouse primary hepatocytes was always more than 98% after either PV or biliary duct (BD)-seeding. After PV-seeding, the portal tree was clogged with injected cells, as observed by gross inspection, and using light and fluorescence microscope evaluation (Fig. 2a). Histological evaluation revealed that the majority of cells remained within the portal branches and only small populations were distributed throughout the parenchyma at 60 h of perfusion culture (Fig. 2b). In contrast to this, cell clusters were observed outside of the biliary tree, 0033 h after BD-seeding (Fig. 2c). Haematoxylin-eosin (H&E) staining showed that a significantly higher proportion of cells was distributed in the parenchyma after BD-seeding (Fig. 2d) compared with the PV-seeding (81.3% versus 20.1%, respectively, p < 0.01) (Fig. 2e). Furthermore, engrafted cells were distributed around central veins (Fig. 2d), which indicated that cells entered the peri-portal area of the parenchyma beyond the barrier of ductule lining ECM, and they subsequently reached the peri-central area across the lobular honeycombed ECM (Fig. 2f).

### Cell source for advanced hepatic function and architecture: adult versus foetal hepatocytes

**Figure 3:**
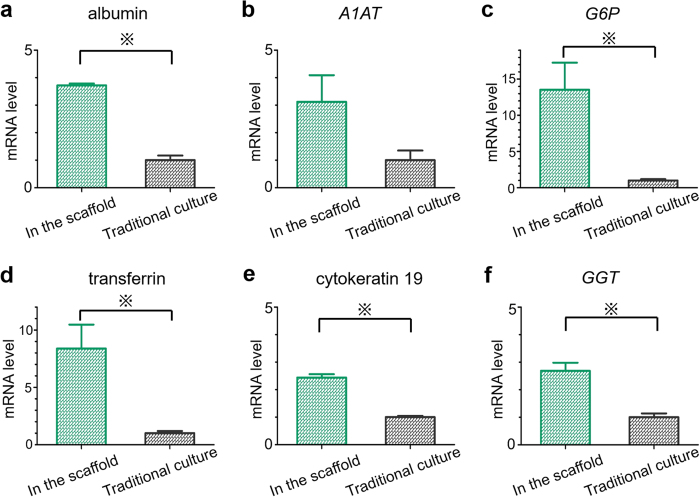
Gene expression analysis. Normalised gene expression levels of albumin (**a**), *A1AT* (**b**), *G6P* (**c**), transferrin (**d**), cytokeratin 19 (**e**), and *GGT* (**f**). All error bars represent standard error of mean (SEM; n = 3) (^※^p < 0.05).

Gene expression analysis was performed to investigate the effect of native liver-derived ECM on foetal cells in the scaffolds, which were seeded through BD and cultivated for 3 days, and the results were compared with gene expression levels in the cells in 2D cultures. The levels of hepatocyte-specific proteins, albumin, alpha-1 antitrypsin (*A1AT*), glucose 6-phosphatase (*G6P*), and transferrin were increased in the scaffold cells (Fig. 3a–d), in comparison with the levels in the cells in 2D cultures, although the difference in *A1AT* level was not significant. Additionally, the levels of cytokeratin 19 and gamma-glutamyl transpeptidase (*GGT*) were higher in the scaffold cells compared with those in the cells in 2D cultures (Fig. 3e,f), which indicates that decellularised liver scaffolds enhance the maturation of foetal hepatocytes into both hepatocyte and cholangiocyte lineages.

**Figure 4:**
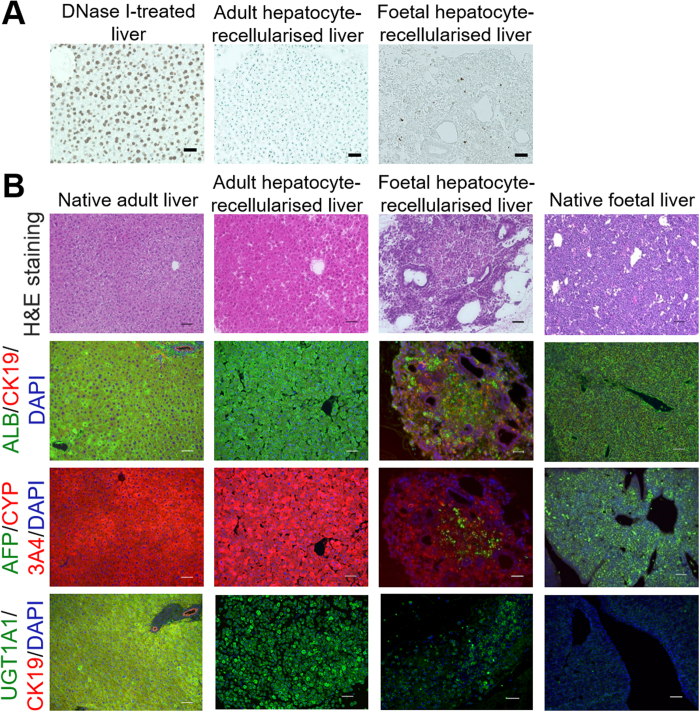
Morphological characterisation of foetal hepatocyte-recellularised liver grafts. (**A**) Quantification of apoptosis by TUNEL staining in DNase I-treated liver sections (positive control), adult hepatocytes, and foetal hepatocytes, showing that less than 5% of the cells were apoptotic at day 2. (**B**) Histological comparison of native and recellularised liver grafts. Left to right: native adult liver, adult hepatocyte-recellularised liver graft (AL), foetal hepatocyte-recellularised liver graft (FL), native foetal liver. Top to bottom: H&E, albumin (ALB, green) and cytokeratin 19 (CK19, red), alfa-feto protein (AFP, green) and CYP3A4 (red), and UGT1A1 (green) and CK19 (red). All sections were counterstained with DAPI (blue). Scale bars: 100 μm (**A**) and 50 μm (**B**).

Immunohistochemical staining of recellularised liver sections was performed at day 2, to evaluate the morphological, functional, and differentiation characteristics of engrafted cells, which were seeded through BD. TUNEL-positive cells were quantified, which showed that less than 5% of the cells were apoptotic in either adult hepatocyte-recellularised liver grafts (AL) or foetal hepatocyte-recellularised liver grafts (FL) (Fig. 4A). The architecture and the expression levels of albumin, a liver-specific protein expressed at the early differentiation stage, and CYP3A4 (cytochrome P450, family 3, subfamily A, polypeptide 4) and UGT1A1 (UDP glucuronosyltransferase 1 family, polypeptide A1), the liver-specific enzymes acquired at late differentiation stage, were similar in both native adult livers and ALs (Fig. 4B), indicating that hepatocyte viability and function were maintained in ALs. Albumin, CYP3A4, and UGT1A1 were expressed in FLs, while only albumin, but not CYP3A4 or UGT1A1, was expressed in the native E14.5 livers (Fig. 4B). Cytokeratin 19-, a cholangiocyte marker, positive cells were observed in FLs, together with the formation of many tubular structures, while its expression was not found in native E14.5 livers. Additionally, alpha-fetoprotein-, an immature hepatocyte marker, positive cells were observed in whole sections of native E14.5 livers, but only in a portion of FLs (Fig. 4B), suggesting that foetal hepatocytes maturated into hepatocytes, and obtained higher liver-enzyme functions, or into cholangiocytes, constructing BDs during cultivation in liver scaffolds.

**Figure 5:**
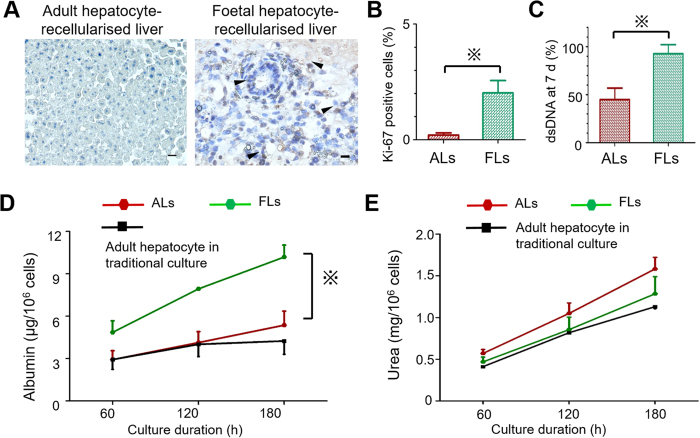
Functional comparison of adult and foetal hepatocyte-recellularised liver grafts. (**A,B**) Quantification of cell proliferation at day 2 using anti-Ki67 staining in adult (ALs) and foetal (FLs) hepatocyte-recellularised liver grafts. (**C**) Quantification of DNA levels at day 7 in ALs and FLs as a surrogate for the viability of engrafted cells. (**D,E**) Metabolic activity assessment of ALs and FLs, demonstrating the higher cumulative albumin production (**D**) and non-inferior cumulative urea synthesis (**G**) of FLs compared with ALs. Scale bars: 20 μm. All error bars represent standard error of mean (SEM; n = 3) (^※^p < 0.05).

The cell proliferation rates of adult and foetal hepatocytes were compared using Ki67 staining at day 2, which demonstrated that a higher proportion of engrafted cells was Ki67-positive in FLs than in ALs (2.03% versus 0.20%, respectively, p < 0.01) (Fig. 5A,B). The viability of the engrafted hepatocytes was assessed by the analysis of AL and FL DNA content at week 1. DNA content in ALs was determined to be 44.9 ± 12.0% of the same number of freshly isolated adult hepatocytes, while in FLs it was 92.7 ± 9.4% of the same number of freshly isolated foetal hepatocytes (p = 0.03) (Fig. 5C), indicating a higher survival capacity of foetal hepatocytes in the scaffolds.

To assess the metabolic activity of recellularised liver grafts, albumin production and urea synthesis were quantified. The cumulative albumin production in FLs during 7-day culture was 10.19 ± 0.85 μg/10^6^ cells, and this was higher than that in ALs (5.37 ± 0.99 μg/10^6^ cells, p < 0.01) and that in adult hepatocytes cultivated in the traditional 2D cultures (4.24 ± 0.94 μg/10^6^ cells, p < 0.001) (Fig. 5D). The cumulative urea synthesis was 1.58 ± 0.14 mg/10^6^ cells in ALs, 1.28 ± 0.21 mg/10^6^ cells in FLs, and 1.13 ± 0.02 mg/10^6^ cells in the traditional culture of adult hepatocytes. No significant differences were observed here (Fig. 5E).

## Discussion

Here, we present a strategy to improve the recellularisation process and further develop the whole-organ regeneration approach initiated with the derivation of decellularised scaffolds^4^,^5^,^7^,^8^,^9^, in order to achieve the reconstruction of clinically relevant liver grafts. Recellularisation should lead to the complete reorganisation of complex liver lobular architecture, consisting of the parenchyma, vasculature, and supporting components, and the refinement of the seeding method and the determination or generation of a clinically available cell source are necessary. To the best of our knowledge, this is the first study presenting an efficient seeding method using the biliary tree, which allows whole-lobular cell distribution, while allowing the portal tree to be used for subsequent reintroduction of endothelial and other non-parenchymal cells. Additionally, we demonstrate that foetal hepatocytes, representing an experimental surrogate for induced pluripotent stem cell (iPSC)-derived liver progenitor cells, integrate biliary structures into liver lobules and provide hepatocytic functions not inferior to the functions of mature hepatocytes, when used as a cell source for whole-liver regeneration.

Based on the previously established decellularisation technique^4^,^5^,^7^, we succeeded in achieving complete decellularisation, while preserving native ECM components and intact vascular networks. Previously established systems for perfusion culture^4^,^5^,^7^ were replicated in this study as well, and their adequacy was demonstrated by the fact that the viability of adult hepatocytes was over 95% at day 2, and they retained hepatocyte enzyme expressions of CYP3A4 and UGT1A1. Additionally, previous recellularisation methods, mainly using PV as a seeding route and primary hepatocytes as a cell source, were modified in order to evaluate BD seeding method, and the use of a different cell source, foetal hepatocytes, for the improvement of morphology and functionality of reconstructed liver grafts.

PV has been widely accepted as a route for the repopulation of the liver parenchyma^4^,^5^,^7^,^8^, and multi-step infusion technique has been recommended for PV-seeding method^4^,^7^ in order to prevent portal clogging, which was observed after single-step infusions^4^. However, several days of perfusion are necessary even when this technique is used, in order to allow the seeded cells to leave the vessels and distribute throughout the parenchyma^4^, which raises a concern about the risk of pressure injury to the intravascular hepatocytes. The evaluation of other seeding routes was necessary, in order to facilitate an efficient cell distribution and integration with further PV reintroduction of multiple types of residing cells. We evaluated cell seeding through BD for the first time, and showed that the engraftment efficacy is more than 95% and parenchymal distribution efficacy higher than 80%, following the BD-seeding. This approach does not require excessive or additional pressure, as required during PV-seeding, for the deposition of cells in the parenchyma, and the lack of pressure injury leads to the high cell viability. The much higher efficiency of parenchymal distribution during BD-seeding, compared with PV-seeding can be explained by the lobular microanatomy, where the liver sinusoid and parenchyma are separated by the space of Disse, whereas biliary canaliculus is composed of hepatocytes, and directly in contact with the parenchyma. Additionally, we found cells distributed around central veins and throughout the lobule after BD-seeding, which suggests that the liver lobules can be entirely repopulated by BD-seeding, regardless of zonation, although a previous study reported areal limitation of the seeding through vena cava, in which the cells were deposited in the peri-central area, and PV-seeding, where the cells were deposited in the peri-portal area^5^. Using our approach, the parenchyma can be recellularised through BD, while the vasculatures can be re-endothelialised through the PV (and the seeding through vena cava and hepatic artery), in contrast to PV-seeding, where the seeded hepatocytes remain within the PV and disrupt the subsequent re-endothelialisation. This integration of seeding routes confirms the concepts presented in previous reports, suggesting that the use of multiple routes may facilitate the spatial arrangement of cells^10^. Based on the obtained results, the evaluation of the cell sources was performed using BD-seeding approach.

The lack of an abundant source of human cells represents a major limitation in the regenerative medicine for liver support, and determining the appropriate cell source is a main challenge for the clinical application of assembled liver grafts. Foetal tissues are considered a potential source of human cells for liver engineering^5^,^11^, but it is currently unclear whether foetal cells can be sufficiently expanded for clinical applications^12^ or whether they can properly differentiate and function. Here, prior to comparing adult and foetal hepatocytes, we assessed whether decellularised liver scaffolds enhance the maturation of foetal hepatocytes, and demonstrated the existence of organ-specific cell–ECM communication, which promotes the maturation of engrafted foetal hepatocytes into both hepatocyte and cholangiocyte lineages, without the addition of any pro-differentiation signals. This agrees with the previous studies that were performed using mouse mesenchymal stem cells (MSC)^13^ and human hepatic stem cells^14^ in liver scaffolds and mouse embryonal stem cells (ESC) in kidney scaffolds^15^,^16^. Immunohistochemical evaluation showed that foetal hepatocytes were mainly immature (alfa-feto protein [AFP]^+^ albumin^+^ CYP3A4^-^ UGT1A1^-^ cytokeratin 19^-^) in the native foetal liver, but they maturated into the two different lineages (AFP^-^ albumin^+^ CYP3A4^+^ cytokeratin 19^-^ and AFP^-^ albumin^-^ CYP3A4^-^ cytokeratin 19^+^), and formed BD-like structures in FLs. These results confirm that multi- or bi-potential precursor liver cells can be used for the reconstruction of complex liver lobular architecture, based on the matrix differentiation signals. The repopulation of the biliary tree with biliary epithelial cells independently from liver parenchymal cell may present an alternative approach, but further investigations are required to develop a technique for the recellularisation of biliary tree and achieve functional cooperation between engrafted hepatocytes and cholangiocytes.

Functional assessment revealed that the cells in FLs have the ability to synthesise a larger amount of albumin and a similar amount of urea, compared with the cells in ALs. Good functional outcomes of FLs, comparable to those of ALs, may be the result of foetal hepatocyte maturation and their subsequent acquiring of advanced hepatocytic functions in the scaffolds. Additionally, the higher proliferation activity and increased viability of the foetal hepatocytes compared with the adult hepatocytes, demonstrated by TUNEL-staining and DNA content quantification, may present the reason for good functional outcomes of FLs. The reproduction of the highly-complex native-liver environment, in which even the mature hepatocytes have unlimited proliferative ability, is currently unattainable in recellularised liver grafts. Therefore, the use of cell types with high proliferative and survival potential *ex vivo* (i.e., immature hepatocytes) is preferred.

This study, demonstrating the potential of foetal hepatocytes, does not necessarily suggest the widespread use of foetal cells, which can raise serious ethical concerns, but it highlights the potential use of iPSC-based whole-organ assembly research^17^. Based on our results, it is possible that iPSC-derived precursor hepatocytes would have similar properties as the foetal hepatocytes, allowing the reconstruction of liver grafts with parenchymal and biliary components and functions non-inferior to that of the mature hepatocytes, through enhanced differentiation. The potential availability of iPSC-derived liver progenitor cells is important, considering the challenges related to the differentiation of iPSCs to mature hepatocytes with functional and regenerative characteristics identical to those of the cells residing in the human liver^18^,^19^. iPSCs also offer the advantages of indefinite propagation in undifferentiated status, supplying their vast quantities, and the potential of differentiation into any cell type required for whole-liver assembly. Additionally, the use of autologous recipient-derived iPSCs allows the assembly of an entirely non-antigenic liver graft, which eliminates the risk of immunological rejection or the need for life-long immunosuppressive therapy. Further investigations of the suitable differentiation stage of iPSC-derived cells that can be seeded in the scaffolds and effective medium that can induce differentiation are necessary, ultimately using clinically relevant quantities of cells in a human sized-liver scaffold.

In addition to the potential clinical use of engineered livers as grafts in liver transplantation therapy, they can be used as 3D culture systems, for the induction of the proliferation and differentiation of various cell types, and *ex vivo* preclinical models for drug metabolism, cell-ECM interactions, and liver development studies, closely imitating complex *in vivo* environment. The deeper understanding of stem cell-ECM niche interactions/stem cell fate decision and liver regeneration may be achieved through the development of recellularised liver grafts with iPSC-derived cell.

In conclusion, the seeding of cells through the biliary tree is a promising approach for efficient repopulation of the parenchyma of liver scaffold. Additionally, foetal hepatocytes represent a good candidate for the cell source for whole-liver assembly, supporting the use of iPSC-derived cells in the future studies.

## Methods

### Animals

Male C57BL/6 mice and C57BL/6 green fluorescent protein (GFP) gene-carrying mice (SLC, Hamamatsu, Japan) were used for hepatocyte isolation, and these cells were used to evaluate cell seeding techniques, hepatocyte function, and for histological studies. Male Lewis rats (250–300 g; SLC) were used for liver harvesting and the preparation of the 3D liver scaffold. All experimental protocols were approved by the Animal Experimentation Committee of Kyoto University and all animal experimental procedures were performed according to the Animal Protection Guidelines of Kyoto University.

### Donor liver harvest

Rats (8 to 12 weeks old) were anesthetised with isoflurane, and they underwent laparotomy. The gastroduodenal and splenic veins were ligated, and the liver was mobilised. Heparin (0.5 U/g of body weight) was administered intravenously. The diaphragm was cut, in order to clamp the aorta in the thoracic cavity and to transect the inferior vena cava. The abdominal aorta was cannulated with a 24-gauge cannula, and 30–40 mL of phosphate-buffered saline (PBS) was injected. The liver was removed, followed by the cannulation of PV with an 18-gauge cannula, and BD with a 24-gauge cannula. The livers were stored in a cell culture dish with PBS solution and frozen until decellularisation.

### Preparation of decellularised whole-liver scaffold

**Figure 6:**
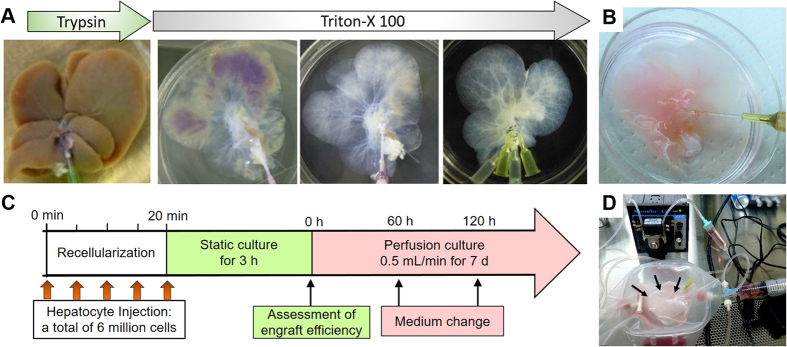
Decellularisation and recellularisation of rat livers. (**A**) Representative images of rat livers during decellularisation process. (**B**) The bile duct of the scaffold was cannulated to inject cells and the culture medium. (**C**) Recellularisation protocol. (**D**) Recellularised liver grafts (arrows) were connected to the recirculation circuit by a cannula inserted into the portal vein and continuously perfused with the culture medium.

The harvested livers of Lewis rats were decellularised as previously described^4^,^7^. Briefly, they were frozen at −80 °C and thawed at room temperature, which was followed by cell-ECM detachment using the perfusion of 0.02% trypsin/0.05% EGTA solution through their PVs for 1 h at 37 °C. Subsequently, 1% Triton X-100/0.05% EGTA solution was perfused for 18–24  h at 1 mL/min for detergent decomposition and solubilisation (Fig. 6A). The samples were washed with PBS for 1 h, and the decellularised liver scaffold was perfused with 0.1% peracetic acid for 2 h, in order to sterilize it.

### Isolation of adult and foetal mouse hepatocytes

Primary adult hepatocytes were obtained from 8- to 12-week-old male C57BL/6 mice, using a modified two-step collagenase perfusion technique, as we described previously^20^. Briefly, mice were anesthetised with isoflurane, laparotomy was performed, and their PVs were cannulated with 25-gauge needles. The livers were then pre-perfused with HBSS-EGTA solution, followed by the perfusion with a collagenase solution containing 0.3% dispase II (Sanko Junyaku, Tokyo, Japan) and 0.3% collagenase type II (Gibco, Palo Alto, CA, USA). Afterward, the livers were excised, minced with scissors, and incubated at 37 °C for an additional 20 min in the collagenase solution. After filtering through the 50 N polypropylene mesh, the suspension was centrifuged three times at 50 × *g* for 4 min. The pellets were collected, and hepatocyte viability was assessed using trypan blue, and determined to be routinely >90%.

Primary foetal hepatocytes were isolated from E14.5 mice livers and were enriched through the formation of cell aggregates, as described previously^21^. Briefly, E14.5 foetal liver tissues were digested by 0.5% collagenase-containing medium and foetal hepatocytes were subjected to the floating culture for about 24 h, in order to form cell aggregates on Petri dishes. They were selected by gravitational sedimentation and the viability was assessed, and confirmed to be >90%.

The cells were suspended at a density of 1.2 × 10^6^ cells/mL for recellularisation, and cultured in collagen-coated dishes (Iwaki, Chiba, Japan) as a control.

### Hepatocyte seeding into the parenchyma of scaffolds

A decellularised liver scaffold was placed in a 60-mm culture dish, and perfused using RPMI 1640 medium through the PV prior to recellularisation. RPMI 1640 (Sigma-Aldrich, St. Louis, MO, USA) was supplemented with 10% foetal calf serum (FCS; ICN, Aurora, OH, USA), 0.0036 μg/mL insulin (MP Biomedicals, Santa Ana, CA, USA), 10 ng/mL epidermal growth factor (EGF; R&D Systems, Minneapolis, MN, USA), 100 U/mL penicillin G and 100 μg/mL streptomycin (Meiji Seika, Tokyo, Japan), and 20 μg/mL gentamycin. A total of 6 × 10^6^ cells was suspended in 5 mL of the culture medium and introduced into the scaffold through either PV or BD, retained after decellularisation (Fig. 6B). At each step, 1 mL of the cell suspension was injected into the scaffold for 1 min, using a peristaltic pump or manually, and the total of five steps was performed in 5-min intervals (Fig. 6C). After 3 h of static culture in an incubator, the perfusate was collected to count the number of cells not retained in the liver scaffolds (Fig. 6C). The engraftment efficiency was calculated as the ratio of the number of cells retained in the liver scaffold to the initial number of seeded cells. The adult hepatocytes of *wild-type* C57BL/6 mice and GFP-positive mice were used for recellularisation, and the comparisons of the two seeding routes. Adult and foetal hepatocytes of *wild-type* C57BL/6 mice were seeded through the BD in order to evaluate the capacity of liver progenitor cells as a cell source for liver recellularisation.

### Perfusion culture of the recellularised liver graft

After 3 h of static culture, we transferred the recellularised liver graft into a customised chamber for perfusion culture. The recellularised liver graft was connected to a recirculation circuit by a cannula inserted into the PV (Fig. 6D) and continuously perfused with RPMI 1640, using a peristaltic pump (Masterflex) at a subphysiological flow rate of 0.5 mL/min (Fig. 6C), in order to prevent the shear stress and minimize the mechanical damage to engrafted cells. The entire perfusion system was placed in a standard CO_2_ (5%) cell incubator at 37 °C. The perfusate was collected every 60 h and replaced with the fresh medium. Histological staining of recellularised sections was performed at day 2 (600’h) of *in vitro* perfusion culture. DNA content was evaluated at day 7 (180 h).

### Histology and immunohistochemistry

Decellularised liver scaffolds and recellularised liver grafts, together with fresh adult and E14.5 foetal mouse livers, were fixed with 4% paraformaldehyde, embedded in paraffin, and cut into 5-μm thick sections. H&E staining and Masson’s trichrome staining were performed according to the standard protocols, to detect any remaining nuclear materials or cellular components in decellularised liver scaffolds. The engraftment of seeded hepatocytes was assessed using H&E staining at 60 h of perfusion culture, by calculating the proportions of cells distributed in the parenchyma to a total number of cells in three randomly selected fields in each recellularised liver graft.

Ki-67 expression in the engrafted cells was analysed immunohistochemically, in order to assess their proliferative capacities. The deparaffinised sections were incubated in 3% hydrogen peroxide in methanol for 10 min to block endogenous peroxidase activity. Sections were incubated at 4 °C for 16 h with monoclonal rat anti-mouse Ki-67 antibody (1:100; eBioscience, San Diego, CA, USA). After the primary antibody had been washed off, the samples were incubated for 60 min with horseradish peroxidase-conjugated anti-rat IgG (Envision Plus Kit; Dako, Glostrup, Denmark) and for 1 min with 3,3′-diaminobenzidine (DAB) substrate (Dako). Counterstaining was performed using haematoxylin. The proportion of Ki-67 positive cells to total cells was calculated in two randomly selected fields in each recellularised liver graft.

TUNEL staining was performed using an *In situ* Apoptosis Detection Kit (Takara Bio, Shiga, Japan) according to the manufacturer’s protocol. The samples were incubated for 1 min with DAB substrate (Dako) and counterstained with methyl green.

Immunofluorescence analysis was performed as previously reported^22^. Antigen retrieval was performed with a Target Retrieval Solution (Dako) and nonspecific binding was blocked with 1.4% bovine serum albumin (BSA; Sigma-Aldrich) dissolved in 0.1% saponin (Wako Pure Chemical Industries) in PBS. The sections were incubated overnight at 4 °C with the following primary antibodies: goat polyclonal anti-mouse albumin (1:200; Bethyl Laboratories, Montgomery, TX, USA), rabbit polyclonal anti-mouse cytokeratin 19 (1:100; Abcam, Cambridge, UK), goat polyclonal anti-mouse alpha-fetoprotein (1:100; Santa Cruz Biotechnology, Santa Cruz, CA, USA), rabbit polyclonal anti-mouse CYP3A4 (1:500; Abcam), rabbit polyclonal anti-mouse UGT1A1 (1:300; GeneTex Inc, Irvine, CA, USA), and goat polyclonal anti-mouse cytokeratin 19 (1:100; Santa Cruz Biotechnology). After washing, the stained sections were incubated with Alexa 488-conjugated donkey anti-goat immunoglobulin (Ig)G (1:500; Invitrogen) and Alexa 555-conjugated donkey anti-rabbit IgG (1:500; Invitrogen), or Alexa 488-conjugated donkey anti-rabbit IgG (1:500; Invitrogen) and Alexa 555-conjugated donkey anti-goat IgG (1:500; Invitrogen) for 2 h at room temperature. Each secondary antibody was diluted at 1:500. After washing, the stained sections were covered with Vectashield mounting medium with 4,6-diamidino-2-phenylindole (DAPI; Vector Laboratories, Burlingame, CA, USA).

All samples were imaged using a fluorescence microscope BZ-9000 (Keyence, Osaka, Japan).

### Vascular tree imaging

The integrity of microvasculature and surface capsule was evaluated by the perfusion of blue dye through the PV and a red dye through the hepatic artery or BD, using peristaltic pump.

### Determination of collagen, glycosaminoglycan, and DNA content

Collagen content was measured indirectly through hydroxyproline content measurements, as described previously^23^, and the glycosaminoglycan content was determined using Blyscan Assay (Biocolor, Ltd., Newtownabbey, UK), according to the manufacturer’s instructions, in order to compare the amount of ECM components in native rat livers and decellularised liver scaffolds. The measured values were normalised to the weight of normal liver. DNA content was measured using Qubit dsDNA HS Assay Kit (Invitrogen, Carlsbad, CA, USA), to ensure the complete removal of native cells from the liver scaffolds. DNA content measurements were used also to assess the viability of engrafted cells in the liver scaffolds, based on the fact that the amount of DNA in each cell remains constant for a given cell type^24^. The DNA levels of livers recellularised with 6 × 10^6^ adult hepatocytes were normalised to the DNA levels of 6 × 10^6^ pre-seeding freshly-isolated adult hepatocytes, while the levels of livers recellularised with 6 × 10^6^ foetal hepatocytes were normalised to those of 6 × 10^6^ pre-seeding foetal hepatocytes.

### Liver function evaluation

Perfusion culture medium samples were collected every 60 h. Albumin content was measured using the mouse albumin ELISA quantitation kit (Bethyl Laboratories), while the urea content was analysed using the QuantiChrom Urea Assay Kit (BioAssay Systems, Hayward, CA, USA), according to the manufacturers’ protocols.

### Quantitative reverse transcription PCR

Total RNA was extracted using a PureLink RNA Mini Kit (Invitrogen), and 100–200 ng of total RNA was reverse transcribed into cDNA using a ReverTra Ace (TOYOBO, Osaka, Japan) according to the manufacturers’ instructions. Primers for the amplification of albumin, *A1AT*, *G6P*, transferrin, cytokeratin 19, *GGT*, and actin-beta (*ActB*) were generated (Supplementary Table S1). Quantitative RT-PCR assay was performed using SYBR-green PCR Master Mix (Applied Biosystems, Foster City, CA, USA) on the ABI 7500 system (Applied Biosystems). Each experiment was performed in triplicate, and the gene expression levels were normalised to the levels of *ActB*.

### Statistical analyses

Data are expressed as mean ± standard error of mean (SEM). We performed statistical analyses using GraphPad Prism for Windows, version 6.0 (GraphPad Software, La Jolla, CA, USA). Statistical significance was defined as p < 0.05. Cell distribution, mRNA expression levels, cell mitosis, and collagen, glycosaminoglycan, and DNA contents were compared using Student’s t-test. The metabolic activity of recellularised liver grafts was compared using repeated-measures analysis of variance (ANOVA), followed by Bonferroni post-tests.

## Additional Information

**How to cite this article**: Ogiso, S. *et al.* Efficient recellularisation of decellularised whole-liver grafts using biliary tree and foetal hepatocytes. *Sci. Rep.* **6**, 35887; doi: 10.1038/srep35887 (2016).

## Supplementary Material

Supplementary Information

## References

[b1] Vacanti J. P. & Langer R. Tissue engineering: the design and fabrication of living replacement devices for surgical reconstruction and transplantation. Lancet 354 **Suppl 1**, SI32–SI34 (1999). 10437854 10.1016/s0140-6736(99)90247-7

[b2] Ott H. C. *et al.* Perfusion-decellularized matrix: using nature’s platform to engineer a bioartificial heart. Nat. Med. 14, 213–221 (2008). 18193059 10.1038/nm1684

[b3] Ott H. C. *et al.* Regeneration and orthotopic transplantation of a bioartificial lung. Nat. Med. 16, 927–933 (2010). 20628374 10.1038/nm.2193

[b4] Uygun B. E. *et al.* Organ reengineering through development of a transplantable recellularized liver graft using decellularized liver matrix. Nat. Med. 16, 814–820 (2010). 20543851 10.1038/nm.2170 PMC2930603

[b5] Baptista P. M. *et al.* The use of whole organ decellularization for the generation of a vascularized liver organoid. Hepatology 53, 604–617 (2011). 21274881 10.1002/hep.24067

[b6] Song J. J. *et al.* Regeneration and experimental orthotopic transplantation of a bioengineered kidney. Nat. Med. 19, 646–651 (2013). 23584091 10.1038/nm.3154 PMC3650107

[b7] Soto-Gutierrez A. *et al.* A whole-organ regenerative medicine approach for liver replacement. Tissue Eng. Part C Methods 17, 677–686 (2011). 21375407 10.1089/ten.tec.2010.0698 PMC3103054

[b8] Yagi H. *et al.* Human-scale whole-organ bioengineering for liver transplantation: a regenerative medicine approach. Cell Transplant. 22, 231–242 (2013). 22943797 10.3727/096368912X654939 PMC3682787

[b9] Fukumitsu K., Yagi H. & Soto-Gutierrez A. Bioengineering in organ transplantation: targeting the liver. Transplant. Proc. 43, 2137–2138 (2011). 21839215 10.1016/j.transproceed.2011.05.014 PMC3156419

[b10] Scarritt M. E., Pashos N. C. & Bunnell B. A. A review of cellularization strategies for tissue engineering of whole organs. Front. Bioeng. Biotechnol. 3, 43 (2015). 25870857 10.3389/fbioe.2015.00043 PMC4378188

[b11] Soto-Gutierrez A., Wertheim J. A., Ott H. C. & Gilbert T. W. Perspectives on whole-organ assembly: moving toward transplantation on demand. J. Clin. Invest. 122, 3817–3823 (2012). 23114604 10.1172/JCI61974 PMC3484436

[b12] Oertel M. Fetal liver cell transplantation as a potential alternative to whole liver transplantation? J. Gastroenterol. 46, 953–965 (2011). 21698354 10.1007/s00535-011-0427-5

[b13] Jiang W. C. *et al.* Cryo-chemical decellularization of the whole liver for mesenchymal stem cells-based functional hepatic tissue engineering. Biomaterials 35, 3607–3617 (2014). 24462361 10.1016/j.biomaterials.2014.01.024 PMC4678102

[b14] Wang Y. *et al.* Lineage restriction of human hepatic stem cells to mature fates is made efficient by tissue-specific biomatrix scaffolds. Hepatology. 53, 293–305 (2011). 21254177 10.1002/hep.24012

[b15] Ross E. A. *et al.* Embryonic stem cells proliferate and differentiate when seeded into kidney scaffolds. J. Am. Soc. Nephrol. 20, 2338–2347 (2009). 19729441 10.1681/ASN.2008111196 PMC2799178

[b16] Bonandrini B. *et al.* Recellularization of well-preserved acellular kidney scaffold using embryonic stem cells. Tissue Eng. Part A 20, 1486–1498 (2014). 24320825 10.1089/ten.tea.2013.0269 PMC4011423

[b17] Handa K. *et al.* Assembly of human organs from stem cells to study liver disease. Am. J. Pathol. 184, 348–357 (2014). 24333262 10.1016/j.ajpath.2013.11.003 PMC3906514

[b18] Kajiwara M. *et al.* Donor-dependent variations in hepatic differentiation from human-induced pluripotent stem cells. Proc. Natl. Acad. Sci. USA 109, 12538–12543 (2012). 22802639 10.1073/pnas.1209979109 PMC3411998

[b19] Si-Tayeb K. *et al.* Highly efficient generation of human hepatocyte-like cells from induced pluripotent stem cells. Hepatology 51, 297–305 (2010). 19998274 10.1002/hep.23354 PMC2946078

[b20] Kita S. *et al.* The protective effect of transplanted liver cells into the mesentery on the rescue of acute liver failure after massive hepatectomy. Cell Transplant., doi: 10.3727/096368916X690999 (2016). 26883767

[b21] Yasuchika K. *et al.* Establishment of a highly efficient gene transfer system for mouse fetal hepatic progenitor cells. Hepatology 36, 1488–1497 (2002). 12447875 10.1053/jhep.2002.36951

[b22] Kawai T. *et al.* Keratin 19, a cancer stem cell marker in human hepatocellular carcinoma. Clin. Cancer Res. 21, 3081–3091 (2015). 25820415 10.1158/1078-0432.CCR-14-1936

[b23] Koyama T. *et al.* Effects of oral intake of hydrogen water on liver fibrogenesis in mice. Hepatol Res. 44, 663–677 (2014). 23682614 10.1111/hepr.12165

[b24] Taguchi T. *et al.* Blockade of RAGE-amphoterin signalling suppresses tumour growth and metastases. Nature. 405, 354–360 (2000). 10830965 10.1038/35012626

