# Supplementary material for: Efficient recellularisation of decellularised whole-liver grafts using biliary tree and foetal hepatocytes
Source: Sci Rep. 2016 Oct 21;6:35887. doi: 10.1038/srep35887 (PMC5073336; doi:10.1038/srep35887)
Supplement: Supplementary Information [file srep35887-s1.doc]

**Efficient recellularisation of decellularised whole-liver grafts using biliary tree and foetal hepatocytes**

Satoshi Ogiso, Kentaro Yasuchika^＊^, Ken Fukumitsu, Takamichi Ishii, Hidenobu Kojima, Yuya Miyauchi, Ryoya Yamaoka, Junji Komori, Hokahiro Katayama, Takayuki Kawai, Elena Yukie Yoshitoshi, Sadahiko Kita, Katsutaro Yasuda, Shinji Uemoto

**Supplementary Table S1**

**Primer sequences for quantitative RT-PCR analysis**

| Gene | Sense primer | Antisense primer |
| --- | --- | --- |
| *Alb* | CGAGAAGCTTGGAGAATATGG | GTCAGAGCAGAGAAGCATGG |
| *A1AT* | TCGATCCTAAGCACACTGAGG | GGCTTGTAAGACTGTAGC |
| *G6P* | TGCATTCCTGTATGGTAGTGG | GAATGAGAGCTCTTGGCTGG |
| *Trf* | CTCTTGGGAAAGACCTGCTG | CTGCCATGACAGGCACTAGA |
| *CK19* | GTGCCACCATTGACAACTCC | AATCCACCTCCACACTGACC |
| *GGT* | ATGAGCTCTGAGTTCTAGGCC | CCACAGCTCTCTTCACATCG |
| *ActB* | TGGAGAAGAGCTATGAGCTGC | GATCCACATCTGCTGGAAGG |

Abbreviation: *Alb*, albumin; *A1AT*, alpha-1 antitrypsin; *G6P*, glucose 6-phosphatase; *Trf*, transferrin; *CK19*, cytokeratin, *GGT*, gamma-glutamyl transpeptidase; *ActB*, actin-beta.
